# The effect of cognitive emotion regulation on direct-acting antivirals adherence in patients with hepatitis C

**DOI:** 10.3389/fphar.2024.1369166

**Published:** 2024-11-05

**Authors:** Adina Turcu-Stiolica, Irina Paula Doica, Bogdan Silviu Ungureanu, Mihaela-Simona Subtirelu, Dan Nicolae Florescu, Razvan-Aurelian Turcu-Stiolica, Ion Rogoveanu, Dan-Ionut Gheonea

**Affiliations:** ^1^ Pharmacoeconomics Department, University of Medicine and Pharmacy of Craiova, Craiova, Romania; ^2^ Gastroenterology Department, University of Medicine and Pharmacy of Craiova, Craiova, Romania; ^3^ Trueman Consulting, Craiova, Romania

**Keywords:** adherence, direct-acting antiviral (DAA), hepatitis C, self-reported questionnaire, cognitive emotion regulation

## Abstract

**Introduction:**

Adherence to direct-acting antivirals (DAAs) could be a predictor of chronic viral hepatitis C (HCV) therapeutic failure. We examined the perceptions of patients receiving DAAs to determine how cognitive factors influence their decision to maintain adherence. Also, we explored the threshold of DAAs adherence for obtaining sustained virologic response (SVR) among patients with HCV, in order to better implement a strategy that improves the DAAs adherence in the future clinical practice.

**Methods:**

A single-arm prospective study was performed. Patients with HCV that started and completed DAAs treatment in the County Hospital of Craiova, Dolj, Romania, were enrolled. Patients’ medication adherence was assessed using the HCV-AD10 questionnaire, and the cognitive emotion regulation was measured with CERQ questionnaire (five positive/adaptive cognitive emotion-regulation domains and four negative/maladaptive domains). Spearman correlation analysis was conducted to explore the relationships between adherence and different factors. ROC-curves were used to evaluate the adherence threshold to achieve SVR. A linear regression model was performed to analyze the primary outcome (DAAs adherence) to be the target variable based on given independent variables (age, treatment duration, severity of HCV, the nine adaptive and maladaptive strategies).

**Results:**

368 patients (mean age: 61 years) with HCV diagnosed 4.05 ± 6.38 (average) years ago were enrolled. Mean (±SD) adherence via HCV-AD10 was 91.51 ± 8.34, and the proportion of the participants achieving SVR was 96%. Patients with an adherence less than 84% (5 patients, 1.36%) was considered nonadherent and they have a high probability of not achieving response (sensitivity and specificity of 83% and 80%, respectively). We obtained significantly higher values of three adaptive strategies between adherent and nonadherent patients following DAAs treatment: in positive refocusing (*p*-value = 0.044), refocus on planning (*p*-value = 0.037), and positive reappraisal (*p*-value = 0.047).

**Discussion:**

The interplay between the three adaptive strategies of the cognitive emotion regulation and the enhancement of DAAs adherence contributes to a more holistic comprehension of patient behavior in the context of HCV treatment. Increasing refocusing and planning using goal setting and assisting patients in establishing specific, achievable goals can be crucial strategies for clinicians aiming to improve adherence among their patients.

## 1 Introduction

Chronic viral hepatitis C (HCV) infection still represents a health problem despite the ongoing national eradication programs in Europe (Huiban et al., 2021). Currently, the HCV patients benefit of several treatment regimens that are successful depending on the virus genotype (Bourlière and Pietri, 2019). The incidence of genotype 1b among the general populace reaches a rate of 4.9%, whereas in Romania, it comprises over 50% of the total HCV infections (Constantin et al., 2020).

The accessibility of pan-genotypic, fixed-dose direct-acting antivirals (DAAs) regimens have streamlined the guidance for treatment protocols, reducing the length of HCV treatment, enhancing tolerance, and dramatically elevating rates of Sustained Virologic Response (SVR) (Chehl et al., 2019). Available recommendations in Romania endorse four distinct combinations of DAAs for hepatitis or compensated cirrhosis for either 8 weeks or 12 weeks: Sofosbuvir/Velpatasvir (SOF/VEL), Glecaprevir/Pibrentasvir (GLE/PIB), Grazoprevir/Elbasvir, and Sofosbuvir/Velpatasvir/Voxilaprevir (SOF/VEL/VOX) (Dietz et al., 2022). In cases of compensated cirrhosis and/or prior treatment failure, a 24-week course of Ledipasvir/Sofosbuvir (LDV/SOF) may be prescribed (Harvoni, 2014). Marincu et al. demonstrated the tolerability and efficacy of DAAs in Romanian patients infected with Type 1b HCV compared to previously used interferon-based therapies (Marincu et al., 2021).

Medication adherence is defined by the World Health Organization as the extent to which a patient’s behavior aligns with the agreed-upon recommendations from a healthcare provider and medication nonadherence causes serious negative impact on clinical and economic outcomes (Agh et al., 2023). DAA adherence was measured using a variety of tools, including the Visual Analog Scale (Burton et al., 2018), patients’ records (Ahmed et al., 2021), pill counts (Burton et al., 2018; Petersen et al., 2016; Campos Fernandez de Sevilla et al., 2019), patient-reported count of remaining tablets (Slevin et al., 2019), medication event monitoring system (MEMS) caps (Petersen et al., 2016), electronic blister pack (Lopes et al., 2023), self-reported questionnaires (Simplified Medication Adherence Questionnaire (Campos Fernandez de Sevilla et al., 2019)), or pharmacy dispensing records (Campos Fernandez de Sevilla et al., 2019).

Poor adherence to new HCV medications is not only associated with negative impacts on healthcare costs and treatment outcomes but may also contribute to the development of resistance to antiviral therapies (Giordano et al., 2018). The Infectious Diseases Society of America and the American Association for the Study of Liver Diseases have recently updated the Hepatitis C Guidance (Bhattacharya et al., 2023) with new recommendations addressing the management of low DAAs adherence (Simon et al., 2021). Recognizing that low medication adherence may contribute to treatment failure (Breckenridge et al., 2017), a new algorithm has been proposed specifically for DAA treatment in cases where patients have missed less or more than 7 days of DAA therapy. The recommended management of DAA nonadherence varies depending on whether the interruptions occur before or after completing 28 days of DAA therapy. In most cases, DAA therapy should be restarted immediately. For example, if a patient missed 10 days within the initially planned 8-week DAA therapy, treatment should be extended to the initially planned 8 weeks plus 10 days. Further comprehensive investigations in clinical practice settings are imperative to explore the correlation between DAA adherence and the achievement of SVR12. These investigations should also aim to identify the critical threshold of adherence below which the attainment of SVR12 is adversely affected.

A patient-centered approach, focusing on adaptive coping strategies, has been demonstrated as necessary to enhance treatment adherence in patients with HCV (Sublette et al., 2015). Given that medication adherence is influenced by patient behaviors and individual behaviors are intricately linked with cognition, our primary objective was to evaluate whether patients with HCV exhibit a distinctive cognitive coping style (adaptive strategies: acceptance, positive refocusing, refocus on planning, positive reappraisal, putting into perspective, or maladaptive strategies: self-blame, rumination, catastrophizing, other-blame) that could significantly impact adherence to Direct-Acting Antivirals (DAAs). Simultaneously, we investigated the threshold of adherence below which DAA treatment proved ineffective among HCV patients in Romania.

## 2 Materials and methods

### 2.1 Study design and population

Our prospective single-center study recruited HCV patients receiving DAAs treatment in the University Clinic of Craiova, Romania, in accordance with the national recommendations (Therapeutic protocol regarding the treatment of hepatitis C, 2020). Inclusion criteria comprised individuals who demonstrated proficiency in verbal communication and the ability to successfully complete designated questionnaires. Prospective participants needed to be adults aged 18 years or older, with a documented history of HCV infection and detectable HCV-RNA. Inclusion was not contingent upon the specific quantitative value of HCV-RNA. Eligibility extended to both treatment-naive individuals and those with prior experience in HCV treatment, including previous regimens involving interferon (INF) or pegylated interferon, with or without concurrent ribavirin (RBV) administration. Participants were enrolled irrespective of their fibrosis stage, encompassing individuals with either compensated or decompensated cirrhosis. The determination of cirrhosis presence or absence was established through imaging modalities such as Fibroscan (de Lédinghen and Vergniol, 2008) or serological methods like FibroMax (Morra et al., 2007).

The inclusion criteria were not restrictive concerning patients co-infected with Hepatitis B Virus (HBV); rather, these individuals underwent simultaneous management for HBV infection concurrently with DAAs treatment and for 12 weeks following the completion of HCV therapy (SVR12). Patients with HBV infection who met the criteria for treatment continued it following the national protocol. However, patients co-infected with HIV or those recently diagnosed with malignant pathologies were excluded from the study.

Following the collection of clinical, laboratory, and virological data, gastroenterologists implemented treatment regimens following the guidelines outlined by the National Health Insurance House/Ministry of Health (Therapeutic protocol regarding the treatment of hepatitis C, 2020). The selected regimens included: 1) Ledipasvir/Sofosbuvir (LDV/SOF), 2) Ombitasvir (OBV) and paritaprevir/ritonavir (PTV/R) in conjunction with dasabuvir (DSV), 3) Sofosbuvir/Velpatasvir (SOF/VEL), or 4) Glecaprevir/Pibrentasvir (GLE/PIB). The choice of treatment regimen was at the discretion of the prescribing physicians, who made decisions based on their clinical expertise and the individual characteristics of the patient. All treatments were fully reimbursed by the National Health Insurance House, with no costs incurred by the patients, according with the new cost-volume-outcome contracts. These contracts are signed between the Romanian National Health Insurance House and the marketing authorization holder of the medicine after the Health Technology Assessment (HTA). Romania has a different HTA procedure based on a scorecard (Radu et al., 2016). Upon enrollment in the study via cost-volume-outcome contract, patients are mandated to undergo the entire treatment regimen. They were eligible to receive the DAA treatment if they agreed to be included in the cost-volume-outcome contract. A patient may discontinue the treatment regimen in the event of adverse reactions. All patients underwent HCV viral load testing 12 weeks after completing the treatment to assess for SVR and the treatment was considered effective if the patient achieved SVR12 (undetectable HCV RNA at 12 weeks post treatment).

The study was approved by the University of Medicine and Pharmacy of Craiova Ethics Committee (approval no. 87/12.02.2020) and all participants provided informed consent before engaging (the consent rate was 100%).

### 2.2 Outcomes

We collected the data to determine possible factors influencing DAAs adherence. The data included 1) demographic characteristics: age, sex, marital status, employment status, education level, environment, income; 2) fibrosis stage, HCV genotype; 3) HCV duration; 4) treatment type and duration; 5) SVR12; 6) comorbidities, inclusive cirrhosis status; 7) DAAs adherence; 8) scores of the cognitive emotion regulation.

**FIGURE 1:**
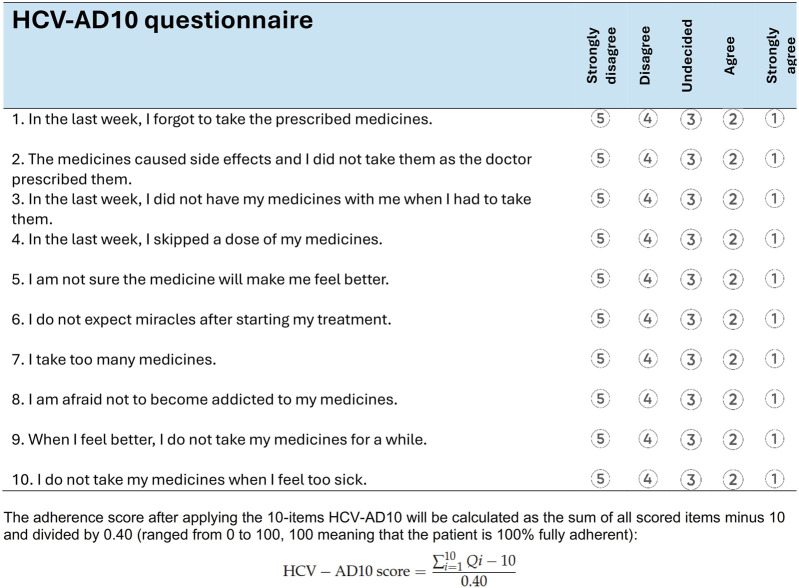
HCV-AD10 questionnaire for measuring the adherence of the direct-acting antivirals among patients with chronic viral hepatitis C (Turcu-Stiolica et al., 2021).

Patients’ medication adherence was assessed using the self-reported HCV-AD10 questionnaire, a tool specifically developed for measuring DAA adherence among patients with HCV in Romania (Turcu-Stiolica et al., 2021). This assessment took place following the completion of their treatment. The treatment duration varied, with 65% of patients receiving an 8-week regimen, 33% undergoing a 12-week regimen, and 2% undergoing a 24-week regimen (Doica et al., 2023). This questionnaire comprises 10 questions with a five-point Likert scale, addressing patient-specific, illness-specific, or medication-specific factors that could influence the DAA adherence (Figure 1). The HCV-AD10 score ranges from 0 to 100, 100 meaning that the patient is 100% fully adherent.

The cognitive emotion regulation was measured with the 36-item Cognitive Emotion Regulation Questionnaire (CERQ). It assesses five positive/adaptive cognitive emotion-regulation domains (acceptance, positive refocusing, refocus on planning, positive reappraisal, putting into perspective) and four negative/maladaptive domains (self-blame, rumination, catastrophizing, other-blame). We used the translated and adapted into the Romanian version (Garnefski et al., 2010) which was concluded for good internal consistency, test-retest stability, and construct validity. Self-blame refers to attributing responsibility to oneself for what one has experienced, while other-blame involves attributing responsibility to the environment or another person. Rumination, also known as focusing on thoughts, is the act of reflecting on the feelings and thoughts associated with a negative event. Catastrophizing involves emphasizing the severity and horror of what one has gone through. Putting into perspective is a coping strategy that minimizes the importance of the event by comparing it to other events, thus emphasizing its relativity. Positive refocusing involves thinking about happy and pleasant things instead of the negative event, whereas positive reappraisal assigns a positive significance to the negative event in terms of personal development. Acceptance is defined as acknowledging and resigning oneself to the reality of what has happened. Refocusing on planning involves considering the steps to take and how to handle the unfavorable event.

### 2.3 Statistical analysis

We performed a descriptive analysis of continuous variables (age, adherence), expressed as mean ± standard deviations (SD), median and interquartile range (IQR), and range (minimum-maximum). Categorical variables (gender, categories of age) were presented as counts and percentages. Additionally, to explore potential correlations between medication adherence and other factors (the nine adaptive and maladaptive strategies), Spearman’s coefficients were calculated and visually presented using heatmaps. To assess differences in characteristics and medication adherence among patients, Kruskal-Wallis H test was employed for continuous variables, and the Chi-square test was used for categorical variables. The ability of varying thresholds of DAAs treatment adherence to predict effectiveness (SVR12, which was measured 12 weeks after completing DAAs treatment) was tested using Receiver Operating Characteristic (ROC) curve analysis to determine the optimal adherence threshold. The area under the curve (AUC) and its 95% confidence interval (95% CI) were computed. When the AUC exceeded 0.6, the optimal cutoff was determined by considering sensitivities and specificities. Sensitivity and specificity were calculated using the ROC curve.

In attempting to clarify the role of emotional regulation factors influencing medication adherence, we performed a multivariate analysis. In our previous study (Doica et al., 2023), we identified age, treatment duration, and the severity of HCV as drivers of DAAs adherence. A linear regression model was performed to analyze the primary outcome (DAAs adherence, as continuous dependent variable) to be the target variable based on the independent variables (age, treatment duration, severity of HCV, the nine adaptive and maladaptive strategies: acceptance (CERQ2), positive refocusing (CERQ4), refocus on planning (CERQ5), positive reappraisal (CERQ6), putting into perspective (CERQ7), self-blame (CERQ1), rumination (CERQ3), catastrophizing (CERQ8), other-blame(CERQ9)) using the stepwise method with forward selection, simultaneously removing those that aren’t important (*p*-value greater than 0.05). The models, which include the predictors after excluding the non-important variables, will be presenting along with their F-statistic and *p*-value.

In order to show how likelihood of being adherent (defined using the threshold derived using the ROC curve analysis) is influenced by cognition, we performed a logistic regression analysis including DAAs adherence as a binary outcome and cognition strategies as continuous predictors.

The afore mentioned statistical analysis was conducted using GraphPad Prism 10.1 (GraphPad Software, Boston, United States), with the significance level set at p less than 0.05 (two-tailed).

## 3 Results

### 3.1 Patients’ characteristics

**FIGURE 2:**
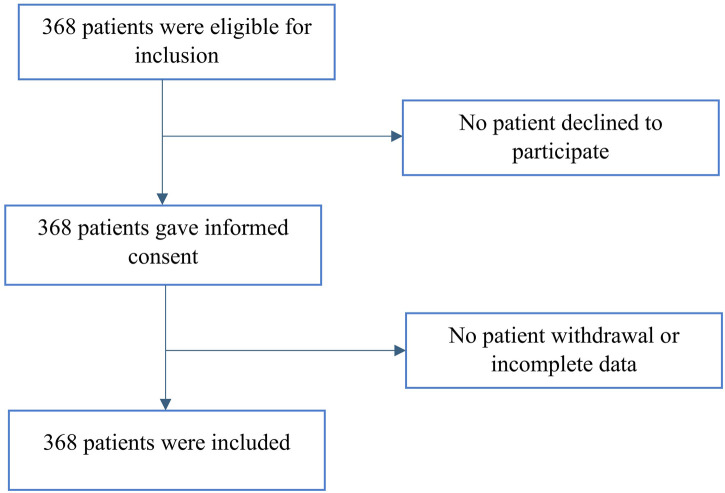
Flow chart of patient inclusion.

During the study period from May 2020 to September 2023, a total of 368 patients diagnosed with HCV approximately 4.05 ± 6.38 (average) years ago were prescribed DAAs (Figure 2), with a predominant representation of females (71.5%). Four patients had HBV co-infection: one patient with an F0 score (no fibrosis in the liver) and three patients with and an F2 score (moderate fibrosis), indicating a low risk of liver-related complications. No adverse reactions were reported in this study, and no patients interrupted the treatment regimen. The baseline characteristics of the patients are detailed in a prior study (Doica et al., 2023). Among them, 105 (28.5%) patients received treatment with LDV/SOF, 142 (38.6%) patients with OBV and PTV/R in conjunction with DSV, 15 (4.1%) patients with SOF/VEL, and 106 (28.8%) patients with GLE/PIB. The SVR was 96% and the mean DAA adherence was 91.51% ± 8.34.

**FIGURE 3:**
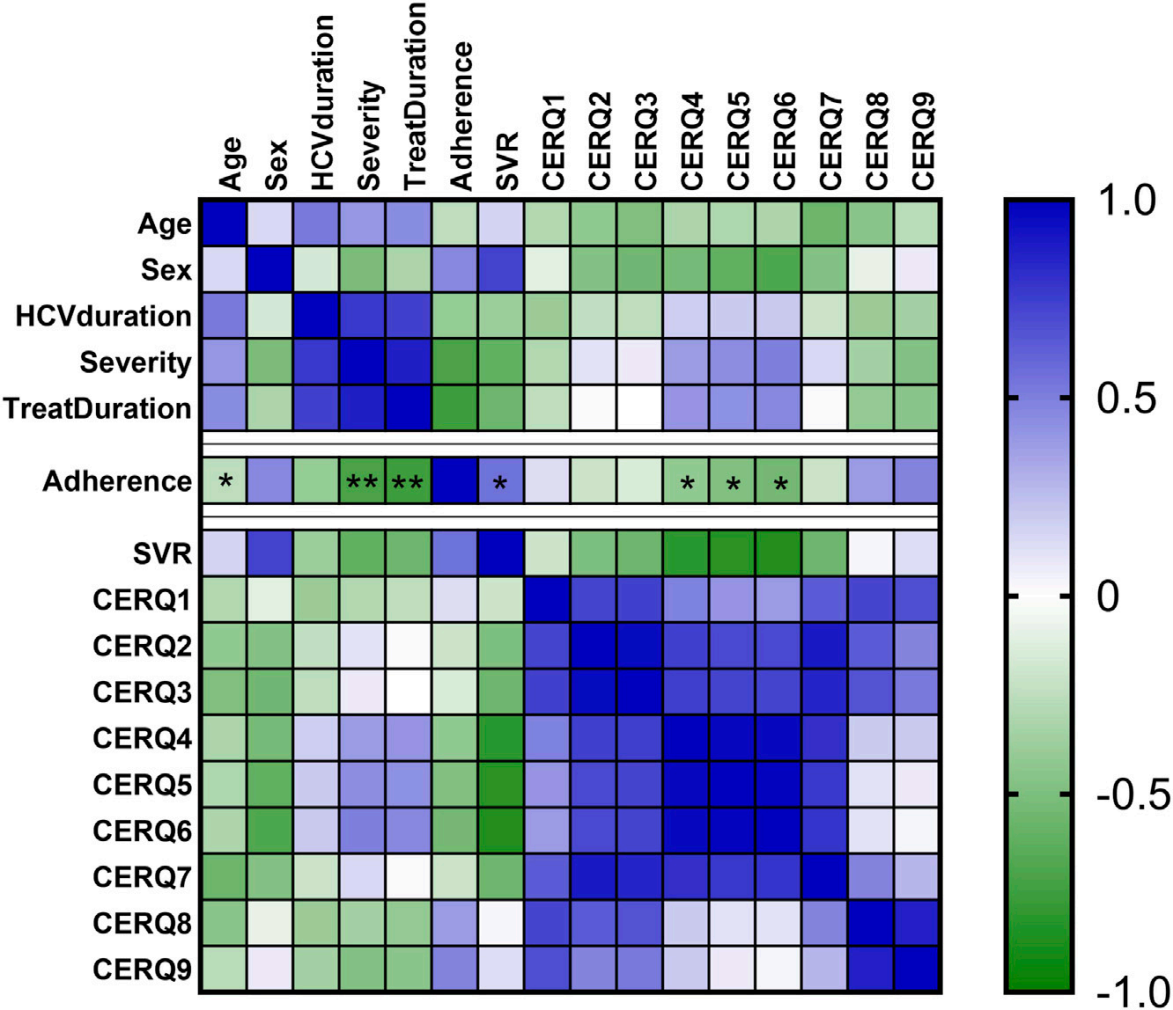
Heatmap of adherence, and CERQ dimensions. The colors from heatmap correspond to the spearman coefficients from negative values (green color) to positive values (blue color). *, *p*-value < 0.05; **, *p*-value < 0.01.

Younger patients were more adherent (rho = −0.112, *p*-value = 0.031). Shorter duration of treatment (rho = −0.101, *p*-value = 0.045) and low severity (rho = −0.167, *p*-value = 0.001) were correlated with high adherence, as in Figure 3.

### 3.2 DAA adherence of the patients

Our study found no association between low DAA adherence and treatment failure (rho = 0.091, *p*-value = 0.08). To assess the DAA adherence threshold, the analyses of the ROC curves were performed, and the AUC (95% CI) was 0.725 (0.455-0.995). The cutoff adherence was 83.75% with a sensitivity of 83% and specificity of 80%. We used this new cutoff adherence, 84% (83.75% was rounded up), to define an adherent/nonadherent patient.

### 3.3 Assessment of the adaptive and maladaptive strategies

**FIGURE 4:**
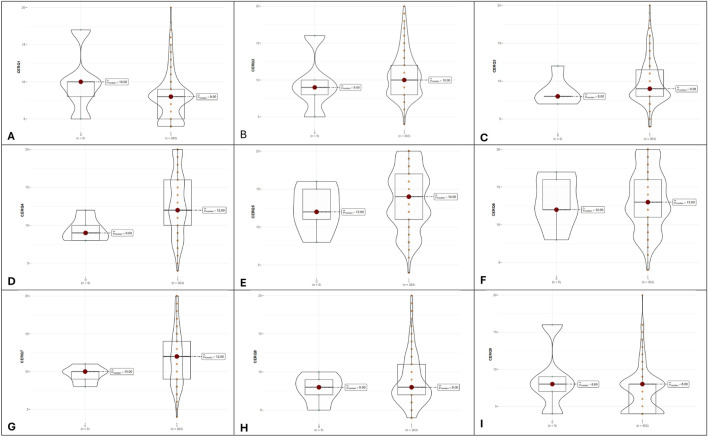
Violin plots illustrating adaptive and maladaptive cognitive strategies between non-adherent (denoted as 0, n = 5) and adherent groups (denoted with 1, n = 363). **(A–I)**, the nine CERQ dimensions.

The nine adaptive and maladaptive strategies evaluated through CERQ questionnaire were compared as in Figure 4. We obtained different values of three adaptive strategies between adherent and nonadherent patients following DAAs treatment. Significantly higher values were obtained for adherent patients in positive refocusing (13.03 ± 4.22 for adherent patients vs. 9.4 ± 1.67 for nonadherent patients, *p*-value = 0.044), refocus on planning (13.78 ± 3.91 for adherent patients vs. 12.4 ± 3.21 for nonadherent patients, *p*-value = 0.037), and positive reappraisal (13.41 ± 3.94 for adherent patients vs. 13.00 ± 3.61 for nonadherent patients, *p*-value = 0.047).

Only three CERQ subscale scores were significantly correlated with DAAs adherence score: positive refocusing (CERQ4), refocus on planning (CERQ5) and positive reappraisal (CERQ6). These correlations were moderate: CERQ4 (rho = −0.422, *p*-value = 0.043), CERQ5 (rho = −0.511, *p*-value = 0.03), and CERQ6 (rho = −0.591, *p*-value = 0.01), as shown in Table 1.

**TABLE 1:** Comparison of adaptive and maladaptive strategies between adherent and nonadherent patients following DAAs treatment.

Score of CERQ mean ± SD median (IQR)	Adherent patients (≥84%) (n = 363)	Non-adherent patients (<84%) (n = 5)	*p*-value
Adaptive strategies			
Acceptance (CERQ2)	10.72 ± 3.02 10 (8–12)	9.6 ± 4.04 9 (6.5–13)	0.368
Positive refocusing (CERQ4)	13.03 ± 4.22 12 (10–16)	9.4 ± 1.67 9 (8–11)	0.044*
Refocus on planning (CERQ5)	13.78 ± 3.91 14 (11–17)	12.4 ± 3.21 12 (9.5–15.5)	0.037*
Positive reappraisal (CERQ6)	13.41 ± 3.94 13 (11–16)	13 ± 3.61 12 (10–16.5)	0.047*
Putting into perspective (CERQ7)	11.83 ± 3.69 12 (9–14)	9.6 ± 1.14 10 (8.5–10.5)	0.168
Maladaptive strategies			
Self-blame (CERQ1)	7.88 ± 3.07 8 (5–9)	10 ± 4.42 10 (6.5–13.5)	0.160
Rumination (CERQ3)	9.92 ± 2.91 9 (8–12)	8.6 ± 1.95 8 (7.5–10)	0.199
Catastrophizing (CERQ8)	8.82 ± 3.3 8 (7–11)	7.8 ± 1.92 8 (6–9.5)	0.688
Blaming others (CERQ9)	7.04 ± 2.93 8 (4–8)	8.8 ± 4.44 8 (5.5–12.5)	0.344

SD, standard deviation; IQR, interquartile range; *, *p*-value < 0.05. Kruskal-Wallis H test was used to compare the two groups of patients.

### 3.4 The role of the cognition factors in DAAs adherence changes

Considering the possible confounding factors (age, treatment duration, and the severity of the disease), we performed the multivariate analysis to assess the role of the emotional regulation factors (Table 2). The severity and CERQ6 were the best group of predictors associated with adherence (F = 7.745, *p*-value = 0.001), explaining 36% of the variance, and no multicollinearity was observed in the second model (Model 2). Greater disease severity was significantly associated with lower adherence (*p*-value = 0.001). Patients who engage in positive reappraisal demonstrate higher adherence (*p*-value = 0.033).

**TABLE 2:** Multivariate analysis of DAAs adherence among HCV patients.

Variable	Unstandardized coefficients	95% Confidence interval	*p*-value	F, *p*-value
Model 1				
Constant	93.93	92.24–95.62	<0.0001	10.829, 0.001
Severity	−0.466	−0.74 to −0.19	0.001	
Model 2				
Constant	96.26	93.53–98.99	<0.0001	7.745, 0.001
Severity	−0.464	−0.74 to −0.19	0.001	
CERQ6	0.296	0.02–0.57	0.033	

Dependent variable: HCV-AD10 score; Method: Stepwise. Model 1’s predictors: (Constant), Severity. Model 2’s predictors: (Constant), Severity, CERQ6.

The general equation to predict DAAs adherence as in Model 2 is:

DAAs adherence=96.26 ‐0.464*Severity+0.296*CERQ6

### 3.5 The influence of cognition on the likelihood of being adherent

Table 3 summarizes the logistic regression analysis, including the nine cognitive factors as predictors. We found a positive association (odds ratio greater than 1) between CERQ 4, CERQ5 and CERQ6 and the outcome (being DAAs adherent). Adherence improves with higher levels of positive refocusing (13.03 vs. 9.4, *p*-value = 0.042) and refocusing on planning (13.78 vs. 12.4, *p*-value = 0.033) improves the adherence. Additionally, better positive reappraisal is associate with improved adherence (13.42 vs. 13, *p*-value = 0.048).

**TABLE 3:** Factors associated with better DAAs adherence among HCV patients.

Variable	OR	95% CI	*p*-value
Self-blame (CERQ1)	0.901	0.803–1.011	0.127
Acceptance (CERQ2)	0.976	0.861–1.107	0.411
Rumination (CERQ3)	1.043	0.916–1.187	0.312
Positive refocusing (CERQ4)	1.043	1.02–1.118	0.042*
Refocus on planning (CERQ5)	1.065	1.015–1.21	0.033*
Positive reappraisal (CERQ6)	1.041	1.011–1.112	0.048*
Putting into perspective (CERQ7)	1.029	0.914–1.159	0.176
Catastrophizing (CERQ8)	1.056	0.941–1.184	0.488
Blaming others (CERQ9)	0.996	0.891–1.114	0.186

Dependent variable: adherent HCV patient. * indicates p < 0.05.

## 4 Discussion

To the best of our knowledge, this study represents the first comprehensive examination of the impact of cognitive emotion regulation on medication adherence, providing insights that can inform adherence-enhancing interventions.

Our findings indicate that high adherence to DAAs in HCV patients is significantly associated with high levels of positive cognitive strategies, including positive refocusing, refocus on planning, and positive reappraisal. In contrast, other authors (Sublette et al., 2015) have reported that fear, stigma, and shame can serve as motivators for completing treatment. These strategies enable patients to cope effectively with treatment challenges and foster resilience. Notably, patients’ motivation to eradicate HCV, along with the associated shame and embarrassment, plays a crucial role in adherence. This aligns with the concept that cognitive emotion regulation contributes to a more comprehensive understanding of patient behavior in HCV treatment.

A multivariate medication adherence model was tested, and adaptive positive reappraisal was the best indicator the DAAs adherence. Patients in our study actively employed adaptive coping strategies to enhance medication adherence, particularly emphasizing positive refocusing on planning. This aligns with findings from another study (Giordano et al., 2018), which emphasized patient engagement and realistic hope for a cure as critical factors influencing adherence to new HCV medications. In discussions with patients, specific timeframes for treatment and a focus on respecting the treatment were considered crucial due to the longer-lasting results on HCV, contributing to bolstered adherence. Moreover, patients attempted to manage medication side effects through adaptive strategies, as lifestyle and behavioral approaches (dietary adjustments with smaller, more frequent meals and light physical activity) or symptomatic relief medications (such as antiemetics for nausea or Antihistamines or topical creams for itching and rashes). Emphasizing the positive outcomes of DAAs, such as no longer being HCV positive after treatment, aligns with the principles of positive refocusing and positive reappraisal (Garnefski et al., 2021), potentially promoting DAA adherence (Spengler et al., 2018).

High medication adherence often requires individuals to manage various stressors related to the disease and the treatment, and the patients may struggle to effectively plan and take action to overcome barriers to adherence (Osborn et al., 2014; Kvarnström et al., 2021). Positive refocusing can lead to better medication adherence by helping individuals maintain a positive outlook and effectively manage adherence challenges. Those who engage in positive reappraisal tend to have better psychological well-being, experience less distress, and demonstrate greater resilience, making them more likely to adhere to their medication regimen despite facing adversity. Individuals who engage in positive reappraisal are more likely to exhibit better psychological well-being and coping with stressors (Lau and Tov, 2023). They may experience less distress and be more resilient in the face of adversity, including adherence-related challenges. A greater tendency to engage in positive reappraisal might demonstrate better medication adherence due to their ability to find positive meaning or outlook in their medication regimen, thus fostering resilience and motivation to adhere to it despite challenges.

These facilitators of DAAs treatment—such as the desire to cure HCV, positive results, and minimal side effects—were similarly identified as determinants of treatment adherence in another study (Patel et al., 2019). The convergence of findings across studies underscores the importance of understanding and leveraging these factors to optimize adherence in patients undergoing HCV treatment.

Another result from the multivariate medication adherence was related to the fact that age, and treatment duration were not identified as facilitators of DAAs adherence, consistent with findings from other studies (Petersen et al., 2016). However, our study demonstrated that younger patients exhibited higher adherence, in contrast to some other studies that found no age-related differences in adherence among HCV patients (Petersen et al., 2016). The influence of treatment duration on DAA adherence was also investigated. Our study observed a decline in adherence over the course of therapy for LDV/SOF, which aligns with previous findings. Specifically, adherence measured by the Medication Event Monitoring System (MEMS) decreased from 98% during weeks 0%–4% to 95% during weeks 8–12 (Petersen et al., 2016).

We demonstrated that the severity of the disease was predictor of DAAs adherence in multivariate analysis. Those who had low severity were more adherent, a finding that has not been demonstrated in other studies in patients with HCV because they did not assess it. Hauber et al. found that patients with moderate or severe fibrosis placed a greater importance on treatment benefit and less on treatment-related side effects than patients with mild or no fibrosis (Brett Hauber et al., 2011).

Previous studies have noted that patients often avoided treatment for HCV due to concerns about side effects, injections, and blood tests (Searson et al., 2014). These findings underscore the multifaceted nature of factors influencing treatment adherence in patients with HCV. These factors were not considered in our study because the patients didn’t experience adverse effects. Educating patients and caregivers about potential side effects of DAA therapy is an essential component of ensuring optimal adherence to DAAs and achieving successful treatment outcomes.

There are minimal data regarding the threshold level of adherence below which the incidence of SVR12 significantly decreases. The conventional threshold commonly used to categorize a patient as adherent is 80%, regardless of the measurement method employed (Pednekar et al., 2019). However, it has been demonstrated that therapeutic failure in HCV with DAAs can be predicted using a lower adherence threshold of 67% (Campos Fernández de Sevilla et al., 2019). Different studies have explored various thresholds, from 80% to 95%, finding no statistically significant differences in achieving SVR between nonadherent and adherent patients (Slevin et al., 2019). Notably, Heo et al. found that higher adherence to combination DAA medications was associated with SVR based on the individual level daily time frame adherence that was determined based on blister pack pop-up dates (Heo et al., 2021). Only one study differentiated the SVR with DAAs therapy based on the severity of HCV. Fabbiani et al. (2021) demonstrated that only 50% patients with F0-F3 disease who took less than 4 weeks of therapy experienced SVR, compared to an SVR of 99% for those who received 4 or more weeks of therapy. Among patients with cirrhosis, a lower SVR12 rate was observed in those who took less than 8 weeks of therapy compared to participants who took 8 or more weeks (83% vs. 95%) (Fabbiani et al., 2021).

Our study identified a unique adherence threshold of 83.75% (84%). It is a slightly higher threshold than usual, but we must consider the rapid reproduction and error-prone nature of the Hepatitis C virus (Pawlotsky, 2016). Also, it is noteworthy that different adherence measurement methods yield varying adherence values. Pharmacy dispensing records were identified as the best indicator of DAA adherence in predicting therapeutic failure compared to pill counts and the Simplified Medication Adherence Questionnaire, with a threshold of 66.66% (Campos Fernández de Sevilla et al., 2019). Another study reported significant differences in DAA adherence values measured with a Visual Analog Scale (VAS) (95.1%) versus an electronic blister pack (76%) (Lopes et al., 2023). Consequently, setting a specific threshold for optimal virologic outcomes requires consideration of the measurement tool used.

Our findings suggest the need for tailored interventions to achieve higher DAAs adherence. A telemedicine program implemented during the COVID-19 pandemic in Romania for HCV therapeutic management resulted in 100% SVR and 100% DAAs adherence (Doica et al., 2021). Previous studies have also highlighted the significant association between adherence and SVR (Cunningham et al., 2020; Heo et al., 2021), supporting other observation that higher DAA adherence is linked with SVR (Akiyama et al., 2019), especially when treating HCV among patients who inject drugs.

Despite providing valuable insights, it’s important to acknowledge the study’s limitations, such as the single-center nature of the study, the small number of nonadherent patients, and the small number of patients which failed to achieve SVR12 (from the statistical perspective), which constrains the ability to perform a multivariate analysis of risk factors for treatment effectiveness related with non-adherence. Since only a small fraction (1.36%) of patients were classified as non-adherent, this small group may not provide sufficient statistical power for robust conclusions about this subgroup. Increasing the overall sample size in future studies could help ensure a more adequate number of non-adherent patients for analysis. Additionally, employing techniques like oversampling non-adherent patients in the data collection phase might help achieve more balanced comparisons. Future studies should aim for a more diverse and representative sample by including patients from multiple centers.

In conclusion, DAAs adherence was high, with no differences between the four regimens. When prescribing the DAA treatment, clinicians should consider age, treatment duration, disease severity, and the facilitators to DAA adherence. This quantitative study enhances our comprehension of patients’ perspectives on adhering to DAAs and has the potential to inform the development of interventions aimed at improving adherence. All patients with incomplete adherence should be asked about the factors contributing to their adherence or nonadherence and counseled on the importance of maintaining adherence. Indeed, increasing refocusing and planning can be crucial strategies for clinicians aiming to improve adherence among their patients. Some practical suggestions can be using goal setting, helping patients set specific, achievable goals related to their treatment plan. Moreover, schedule regular check-ins with patients to discuss their progress and address any challenges they may be facing can be beneficial. These check-ins can help refocus their attention on their treatment plan and provide additional support when needed. A longitudinal design could be employed in future studies. Tracking patients over time would provide a clearer picture of how changes in cognitive strategies impact adherence and whether interventions that enhance these strategies can lead to sustained improvements in adherence. By incorporating these strategies into their practice, clinicians can help patients improve their refocusing abilities and develop effective planning skills to enhance adherence to their treatment plans.

## Data Availability

The raw data supporting the conclusions of this article will be made available by the authors, without undue reservation.
